# Development of extended pharmacokinetic models for propofol based on measured blood and brain concentrations

**DOI:** 10.1038/s41598-024-56863-z

**Published:** 2024-03-15

**Authors:** Masayoshi Kawata, Atsushi Yonezawa, Yohei Mineharu, Kotaro Itohara, Toshiyuki Mizota, Yoshihiro Matsui, Takayuki Kikuchi, Yukihiro Yamao, Etsuko Yamamoto Hattori, Miho Hamada, Daiki Hira, Keiko Furukawa, Susumu Miyamoto, Tomohiro Terada, Kazuo Matsubara, Yoshiki Arakawa

**Affiliations:** 1 Department of Clinical Pharmacology and Therapeutics, Kyoto University Hospital, 54 Shogoin Kawahara-cho, Sakyo-ku, Kyoto, 606-8507 Japan; 2 Graduate School of Pharmaceutical Sciences, Kyoto University, 46-29 Yoshida Shimo-Adachi-cho, Sakyo-ku, Kyoto, 606-8501 Japan; 3 Department of Neurosurgery, Kyoto University Graduate School of Medicine, 54 Shogoin Kawahara-cho, Sakyo-ku, Kyoto, 606-8507 Japan; 4 Department of Artificial Intelligence in Healthcare and Medicine, Kyoto University Graduate School of Medicine, 54 Shogoin Kawahara-cho, Sakyo-ku, Kyoto, 606-8507 Japan; 5 Department of Anesthesia, Kyoto University Hospital, 54 Shogoin Kawahara-cho, Sakyo-ku, Kyoto, 606-8507 Japan; 6 Cancer Center, Kyoto University Hospital, 54 Shogoin Kawahara-cho, Sakyo-ku, Kyoto, 606-8507 Japan

**Keywords:** Pharmacokinetics, Predictive markers

## Abstract

Propofol’s pharmacokinetics have been extensively studied using human blood samples and applied to target-controlled infusion systems; however, information on its concentration in the brain remains scarce. Therefore, this study aimed to simultaneously measure propofol plasma and brain concentrations in patients who underwent awake craniotomy and establish new pharmacokinetic model. Fifty-seven patients with brain tumors or brain lesions who underwent awake craniotomy were sequentially assigned to model-building and validating groups. Plasma and brain (lobectomy or uncapping margins) samples were collected at five time-points. The concentration of propofol was measured using high-performance liquid chromatography. Population pharmacokinetic analysis was conducted through a nonlinear mixed-effects modeling program using a first-order conditional estimation method with interactions. Propofol’s brain concentrations were higher than its plasma concentrations. The measured brain concentrations were higher than the effect site concentrations using the previous models. Extended models were constructed based on measured concentrations by incorporating the brain/plasma partition coefficient (K_p_ value). Extended models showed good predictive accuracy for brain concentrations in the validating group. The K_p_ value functioned as a factor explaining retention in the brain. Our new pharmacokinetic models and Kp value can predict propofol’s brain and plasma concentrations, contributing to safer and more stable anesthesia.

## Introduction

Propofol is widely used for the induction and maintenance of anesthesia during surgery. Real-time analysis of the achieved concentrations is not possible during the maintenance of anesthesia with intravenous propofol infusion. Target-controlled infusion (TCI) systems programmed with pharmacokinetic (PK) and pharmacodynamic (PD) models have been developed to assist with the intravenous administration of propofol^1,2^. Multi-compartmental PK models mathematically describe the distribution and elimination processes of propofol. The PD component describes the relationship between the concentration of propofol at the site of action (effect site) and the clinical effect. During model development, PD parameters are best estimated from synchronous measurements of the plasma concentration and measures of the clinical effect in the same group of subjects^1^. The Marsh model^3^ and Schnider model^4,5^ for propofol are well-established models commonly used globally. Although the PK of anesthetic drugs including propofol has been extensively studied using blood samples from humans, less information is available on the brain^3–12^.

The plasma concentration of intravenous anesthetic drugs after a bolus peak is virtually instantaneous; however, the peak effect of the drug occurs later when the brain concentration equilibrates with that in the central compartment (plasma). The Marsh model was an original PK model established based on data from children^3^, and the effect-compartment equilibrium rate constant (k_e0_ = 0.26 min^−1^) study was later added to the original Marsh model^13^. The k_e0_ was estimated from the electroencephalographic (EEG) response to propofol^6^. Other studies had considered PK-PD models based on the bispectral index^7,14^. In addition, one study developed a PK model for propofol in mice that quantitatively described propofol distribution into and elimination out of the brain^15^. However, brain concentrations have not been used to establish a human PK model for propofol^8,12^.

During awake craniotomy for brain tumor resection, propofol is administered using TCI based on the plasma and effect-site concentrations calculated using the PK-PD model. Precise regulation of the effect-site concentration of propofol is especially crucial in awake craniotomy because rapidly achieving good arousal is essential to identify the specific location of the eloquent cortex, which is the goal of awake craniotomy. However, there is wide interindividual variability in the time until intraoperative awakening, even when TCI is used^16,17^. Establishment of a PK model using measured propofol concentrations aims to identify the causes of individual differences in PD. This is the first step toward solving the problems of the PK-PD model. Thus, it is important to first show actual brain concentrations to find other factors that can explain the wide interindividual variability.

Therefore, this study aimed to develop extended pharmacokinetic models for propofol based on measured plasma and brain concentrations in patients who underwent awake craniotomy. A PK model was established using measured plasma and brain propofol concentrations to accurately describe brain concentrations. This is the first step toward solving the problems of the PK-PD model.

## Results

### Patient characteristics

A total of 105 plasma and 80 brain concentration samples from 29 patients were included in the model-building analysis. Patient characteristics are summarized in Table S1. Furthermore, 51 plasma and 32 brain concentration samples from 28 patients were used to verify the model. Fifty-three cases were diagnosed as glioma, 2 cases as epilepsy, and 2 cases as cavernous hemangioma. There were no significant intergroup differences in patient characteristics.

### Measured and predicted plasma and brain concentrations of propofol

**Figure 1:**
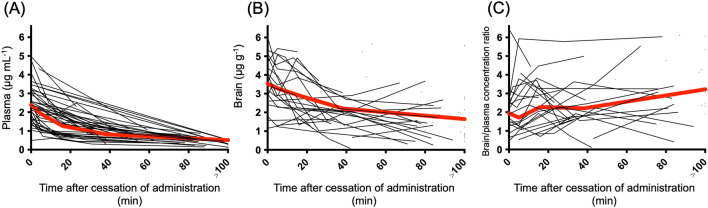
Plasma (**A**) and brain (**B**) concentrations and the ratio of brain/plasma concentration (**C**) of propofol after termination of infusion. Propofol concentrations are measured in all 57 patients. For each patient, plasma and brain samples (5–30 mg) were collected at five time-points: immediately before and 5, 15, 25, and 75 min after the cessation of propofol administration. The gray line shows the data for each patient, and the red line represents the average value.

The measured plasma and brain concentrations of the 57 patients after cessation of propofol administration are shown in Fig. 1. Plasma and brain concentrations gradually decreased over time. The brain/plasma concentration ratio also increased.

**Figure 2:**
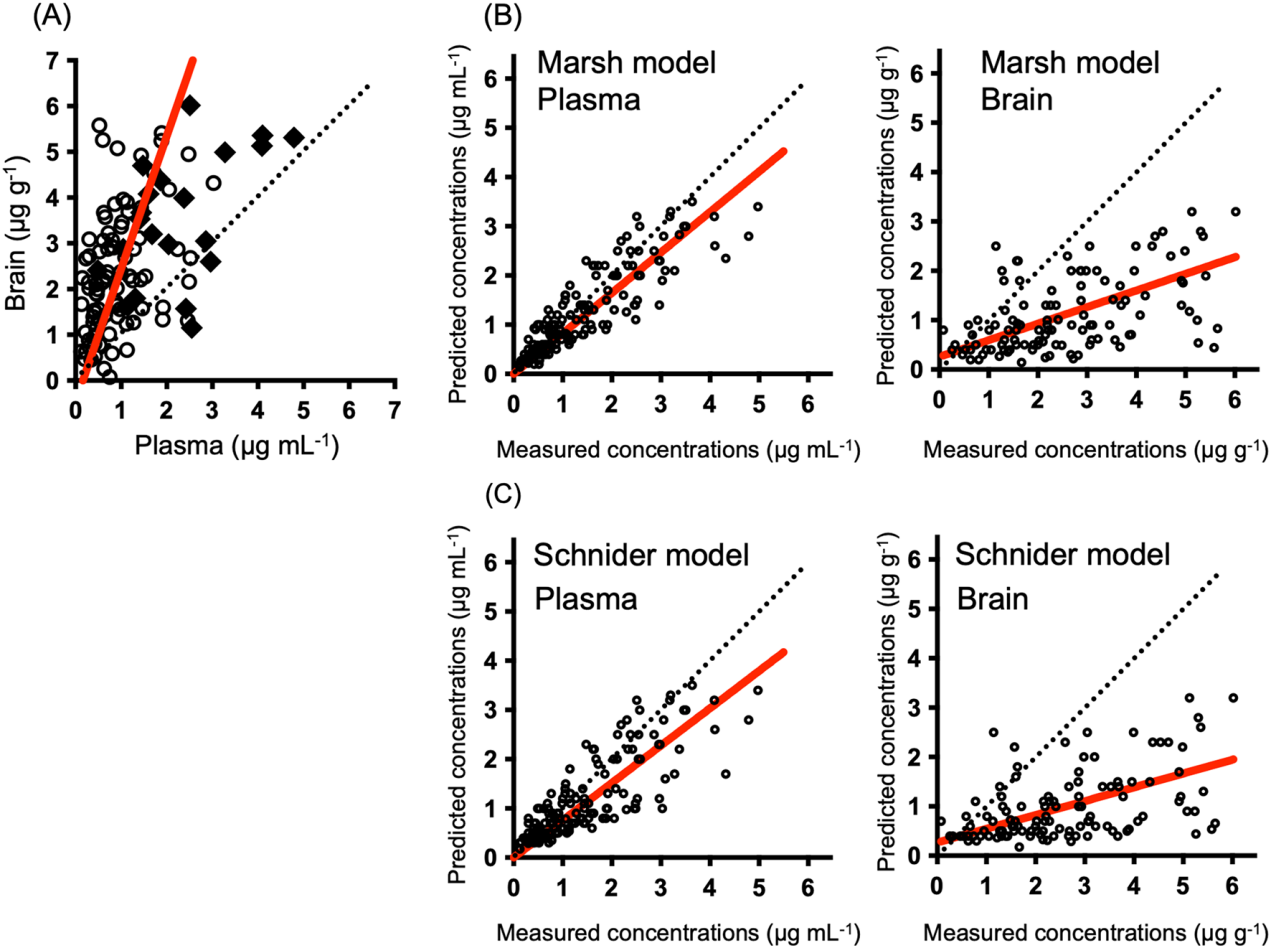
Correlations between measured plasma and brain concentrations (**A**) and correlation between measured and predicted propofol concentrations in plasma and brain (**B**,**C**). There were 156 plasma concentrations and 112 brain concentrations from 57 patients. Correlations between measured plasma and brain concentrations at all points are shown (**A**). Measured plasma and brain concentrations at steady state points are shown as filled diamonds. Other measured concentrations are shown as empty circles. Predicted concentrations are shown based on the Marsh model (**B**) and Schnider model (**C**) and the measured concentrations in the plasma and brain. The red line represents the regression line.

The relationship between the measured and predicted concentrations of propofol in 57 patients is shown in Fig. 2. The measured brain concentrations were higher than the plasma concentrations. Similar results were also obtained at steady state points, which were only points before the cessation of administration (Fig. 2A). There was a good correlation between the measured plasma concentrations and the predicted concentrations based on the Marsh model^3^ and Schnider model^4,5^. However, the measured concentrations in the brain were higher than the predicted concentrations. There was a poor correlation between the predicted and measured brain concentrations in full data (Fig. 2B,C).

### Population pharmacokinetics (PopPK) modeling based on the Marsh model

The final model structure is shown in Supplementary Fig. S1A. A three-compartment with effect-compartment models were selected, corresponding to the Marsh model^3^. As shown in Fig. 2, the Marsh model^3^ accurately predicted plasma concentrations of propofol. We determined that the existing model has adequate predictive ability for plasma concentration, and the mean values of k_10_, k_12_, k_13_, k_21_, k_31_, and the central volume of distribution (V_1_) were fixed with the typical parameter values used in the Marsh model^3^. The parameters involved in the effect compartment and inter and intra individual variabilities were estimated. Interindividual variability, which did not improve the model, was excluded from the final model. For residual variability, the proportional and additive error models provided a better model fit for plasma and brain concentrations, respectively. For the predicted concentrations of propofol in the brain, the inclusion of a K_p_ value for k_1e_ improved the model fitting, where K_p_ is the partition coefficient corresponding to the ratio of the brain to the plasma propofol concentration at steady state, k_1e_ is the distribution rate constant from the central compartment to the brain compartment.

The final estimates of propofol PopPK parameters are presented in Table 1. Of all the covariates examined, we did not find any improvement in the predictability of the model by adding covariates (Supplementary Table S2, Supplemental Digital Content).

**Table 1:** Population pharmacokinetic parameters of propofol (Extended Marsh model).

Parameter	Model-building			Bootstrap results (N = 500)		
	Estimates	%RSE		Median	95% CI	
k_10_ (min^−1^)	0.119		Fixed	0.119		Fixed
k_12_ (min^−1^)	0.114		Fixed	0.114		Fixed
k_13_ (min^−1^)	0.0419		Fixed	0.0419		Fixed
k_21_ (min^−1^)	0.055		Fixed	0.055		Fixed
k_31_ (min^−1^)	0.0033		Fixed	0.0033		Fixed
V_1_ (L kg^−1^)	0.228		Fixed	0.228		Fixed
k_1e_ (min^−1^)	0.014	34.8		0.014	0.0069 − 0.034	
K_p_	1.30	8.92		1.33	1.08 − 1.60	

Interindividual variability, CV%	Variance	%RSE	Shrinkage	Variance	95% CI	
IIV for k_10_	18.8	27.6	11.9	18.6	13.1–23.2	
IIV for k_13_	59.7	50.6	36.0	62.3	21.6–87.0	
IIV for K_p_	40.7	52.5	17.6	37.8	16.9–59.4	

Residual variability	Variance	%RSE	Shrinkage	Variance	95% CI	
Proportional error for plasma, CV%	19.6	28.6	15.9	19.1	13.5–24.5	
Additive error for brain, μg/g	0.869	24.1	13.5	0.860	0.655–1.10	

k_1e_ = Q_4_/V_1_.

*95% CI* 95% confidence interval, *CV* coefficient of variation, *IIV* interindividual variability, *RSE* relative standard error, *Q4* inter-compartmental clearance between the central and brain compartments.

### PopPK modeling for elimination phase description

The final model structure is shown in Supplementary Fig. S1B. A two-compartment with effect-compartment model was selected. The parameters involved in the effect compartment and inter and intra individual variabilities were estimated. Interindividual variability did not improve the model; therefore, it was excluded from the final model. For residual variability, the proportional and additive error models provided a better model fit for plasma and brain concentrations, respectively. For the predicted concentrations of propofol in the brain, the inclusion of a K_p_ value for k_1e_ improved the model fitting.

The final estimates of propofol PopPK parameters are presented in Table 2. Of all the covariates examined, no improvement in the model’s predictability was observed by adding covariates.

**Table 2:** Population pharmacokinetic parameters of propofol (New model).

Parameter	Model-building		Bootstrap results (N = 500)			
	Estimates	%RSE	Median	95% CI		
CL_1_ (L min^−1^)	1.78	5.22	1.79	0.229 − 1.99		
V_1_ (L)	41.1	17.7	39.7	19.4 − 55.1		
CL_2_ (L min^−1^)	0.806	11.6	0.871	0.661 − 2.13		
V_2_ (L)	123	26.5	117	60.5 − 2271		
k_1e_ (min^−1^)	0.0149	35.8	0.0147	0.00615 − 0.0392		
K_p_	1.39	9.78	1.40	1.13 − 1.82		

Interindividual variability, CV%	Variance	%RSE	Shrinkage	Variance	95% CI	
IIV for CL_1_	16.2	19.8	12.0	15.4	8.97–84.9	
IIV for K_p_	38.5	26.0	19.4	35.7	15.1–56.6	

Residual variability	Variance	%RSE	Shrinkage	Variance	95% CI	
Proportional error for plasma, CV%	21.2	8.68	9.87	20.7	16.8–24.5	
Additive error for brain, μg/g	0.879	11.9	13.03	0.865	0.649–1.094	

k_10_ = CL_1_/V_1_, k_12_ = CL_2_/V_1_, k_21_ = CL_2_/V_2_.

*95% CI* 95% confidence interval, *CV* coefficient of variation, *IIV* interindividual variability, *RSE* relative standard error.

### Model evaluation

**Figure 3:**
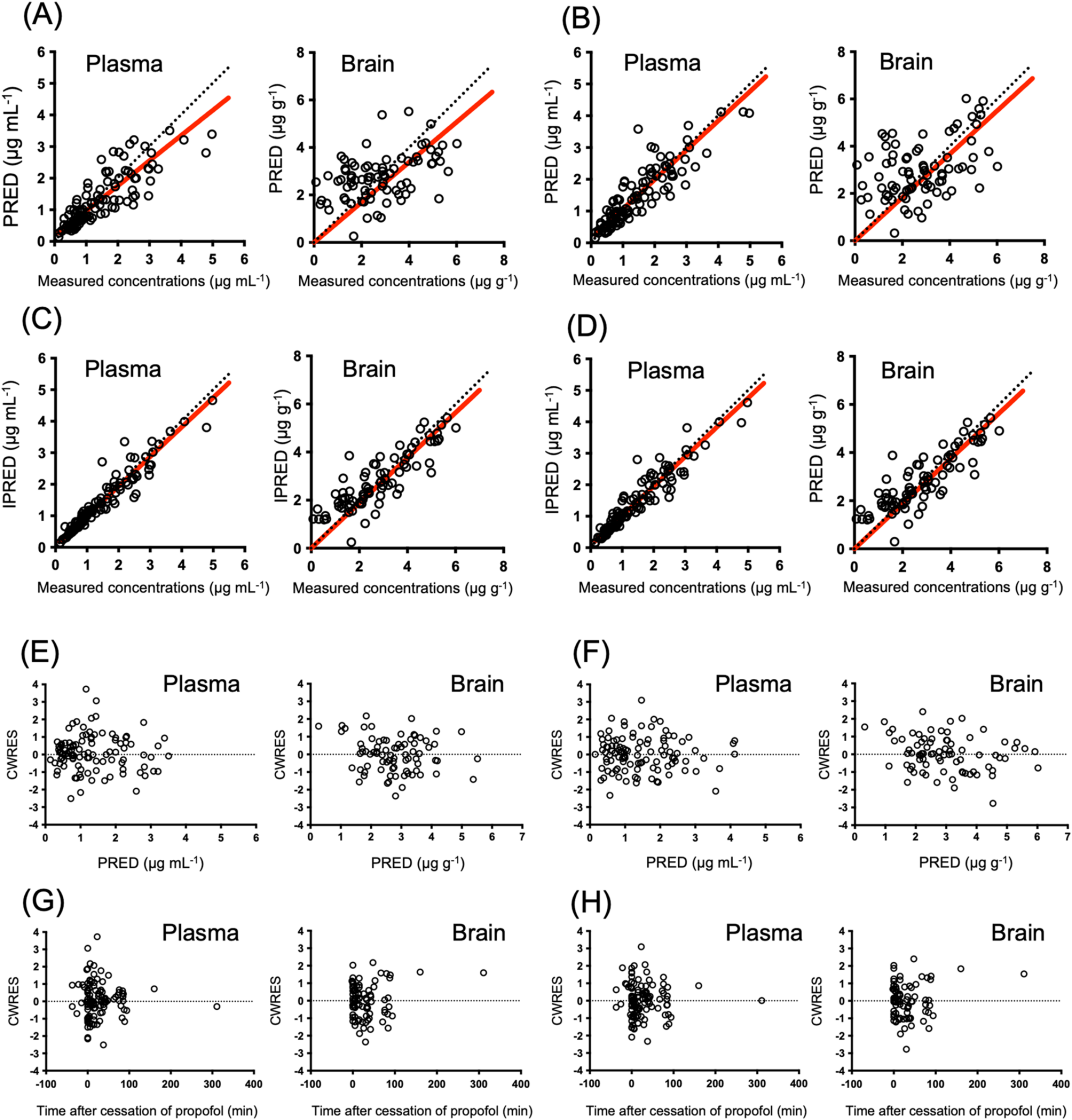
Goodness-of-fit plots for the final propofol model. Measured concentrations versus population predictions (PRED) and individual predictions (IPRED) are shown in (**A**) and (**B**), (**C**) and (**D**), respectively. Conditional weighted residuals (CWRES) versus PRED versus time after cessation of propofol are shown in (**E**) and (**F**), (**G**) and (**H**). (**A**), (**C**), (**E**), (**G**) are shown in our extended Marsh model. (**B**), (**D**), (**F**), (**H**) are shown in our new model. Empty circles represent patient data in model-building populations. There are 106 plasma concentrations and 80 brain concentrations in the 29 patients. The red line represents the regression line.

**Figure 4:**
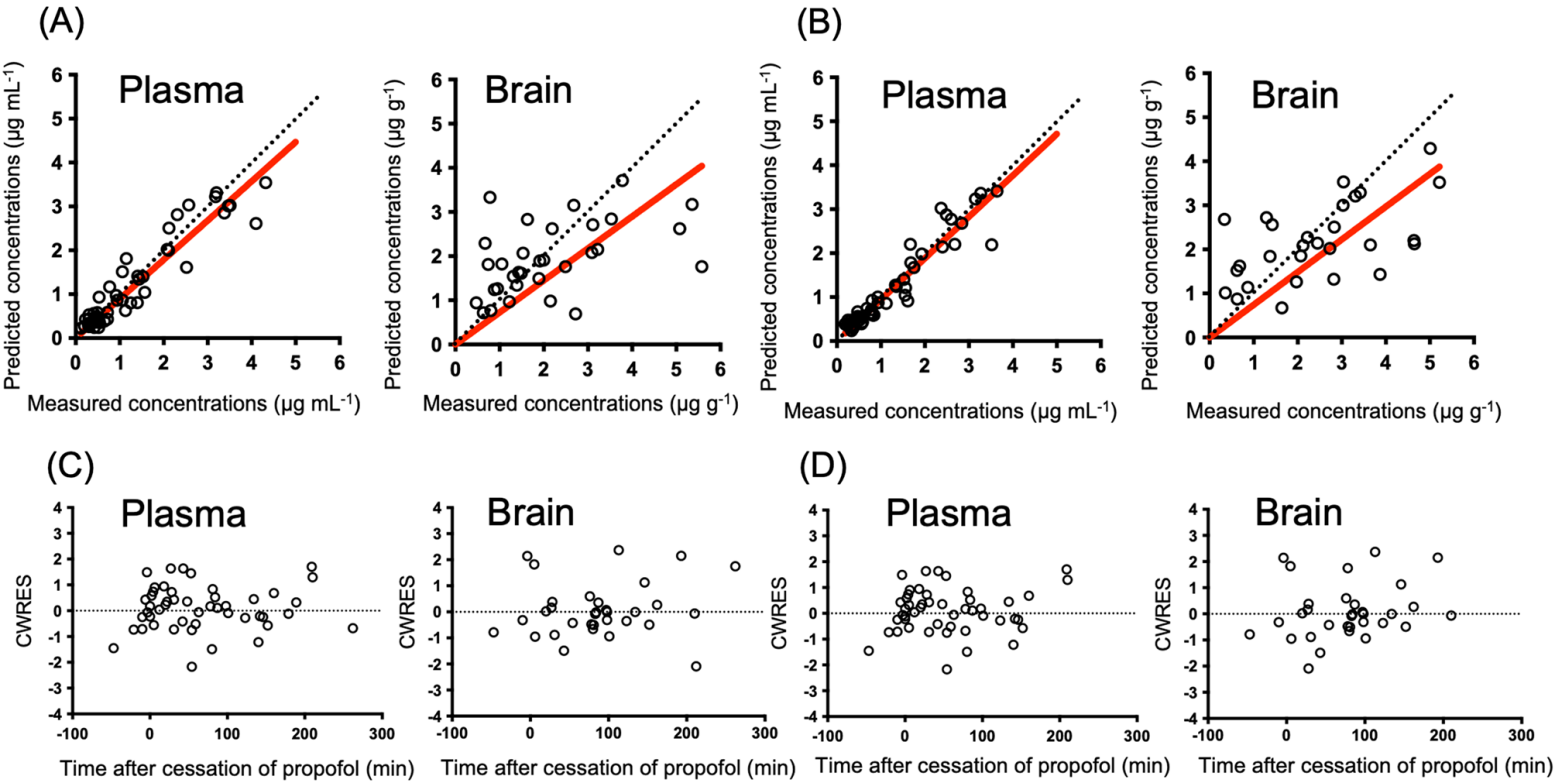
Comparison of measured and predicted concentrations of propofol in the validating populations. Measured concentrations versus those predicted using the extended model (**A**) and the new model (**B**) shown as Empty circles represent patients’ data in the validating populations. Conditional weighted residuals (CWRES) versus time after cessation of propofol are shown in (**C**) and (**D**). There were 51 plasma concentrations and 32 brain concentrations in 28 patients. The red line represents the regression line.

The goodness-of-fit plots for the final model are shown in Fig. 3. The plots of the measured population-predicted value (PRED) and individual predicted value (IPRED) revealed a favorable agreement between the model predictions and observations of patients in the model-building populations. Moreover, conditional weighted residuals (CWRES) were evenly distributed around zero against PRED and the time after propofol cessation. A comparison of the measured and predicted concentrations from our extended and new models in the validating population is shown in Fig. 4. The median (range) performance error (MdPE) values from the extended model for the plasma and brain concentrations of propofol for the validating data were 4.5% (range: − 48.6 to 113%) and − 3.2% (− 59.0 to 293%), respectively. The mean (range) absolute performance error (MdAPE) values from the extended model were 20.5% (1.8 to 113%) and 28.1% (1.0 to 293%) for the plasma and brain concentrations, respectively. The median (range) MdPE values from our new model for the plasma and brain concentrations of propofol for the validating data were 4.76% (range: − 51.6 to 76.4%) and 1.01% (− 166 to 170%), respectively. The mean (range) MdAPE values from our new model were 16.3% (0.59 to 76.4%) and 48.3% (1.54 to 170%) for the plasma and brain concentrations, respectively.

**Figure 5:**
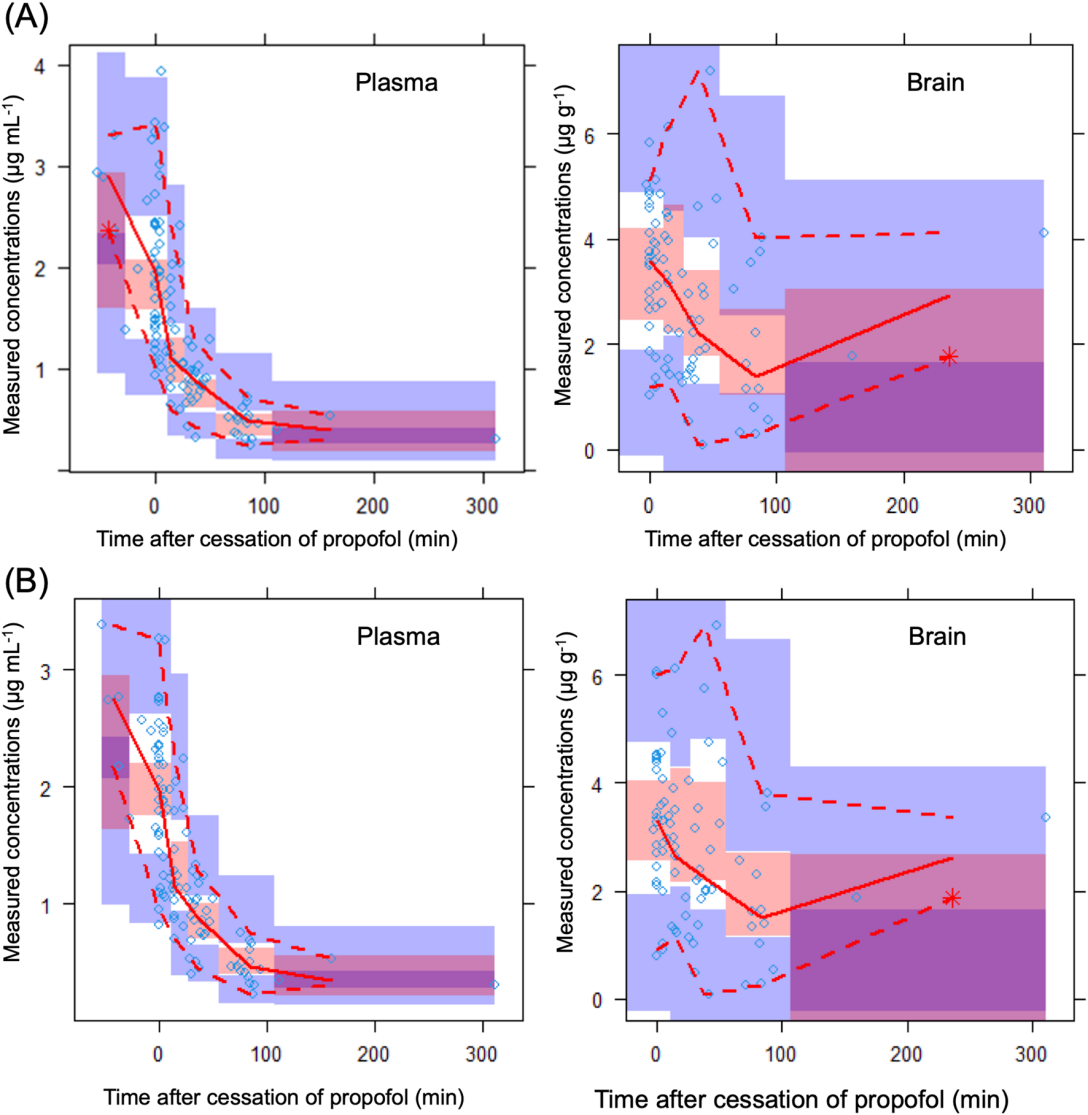
Prediction-corrected visual predictive check plots using plasma and brain-predicted values for propofol. Each symbol represents the measured concentration. The solid lines in each graph denote the 5th, 50th, and 95th percentiles of the measured concentrations. The dotted lines in each graph represent the 5th, 50th, and 95th percentiles of the predicted concentrations based on the extended model (**A**) and the new model (**B**). The shaded areas indicate the simulation-based 95% CI for the 5th, 50th, and 95th percentiles.

**Figure 6:**
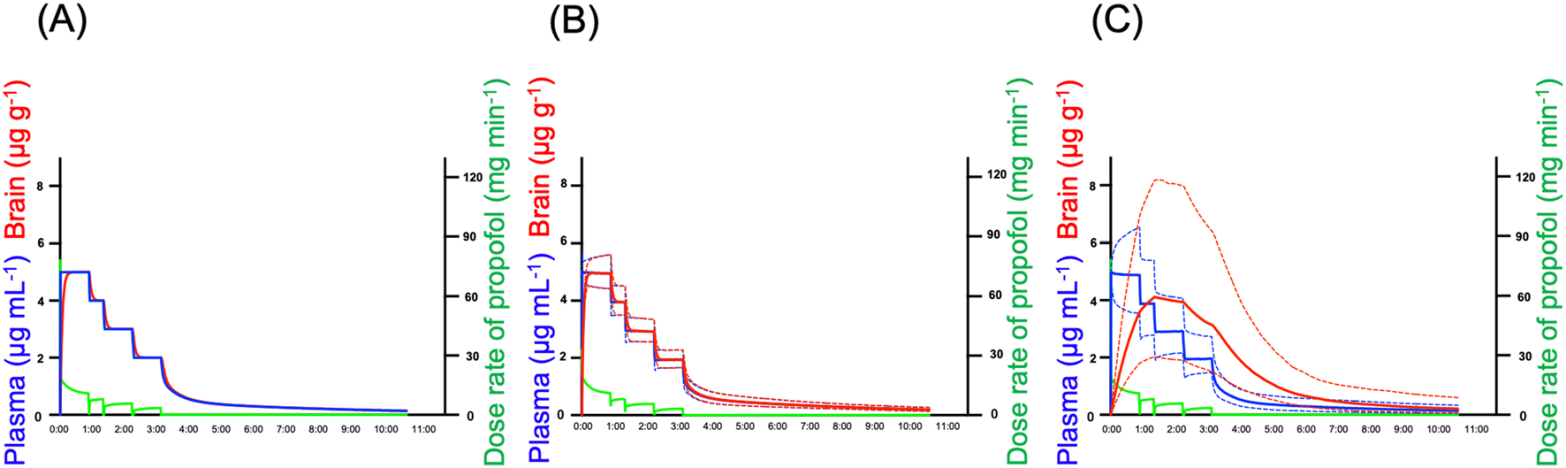
Predicted concentrations of propofol using Marsh (**A**), Schnider (**B**), and extended models (**C**) in a typical propofol dosing schedule. The predicted plasma and brain concentrations based on the Marsh model (**A**), Schnider model (**B**), and extended model (**C**) from a typical example propofol dosing schedule (when propofol administration is completed at 3 h and 30 min) are shown. Blue and red solid lines represent the predicted plasma and brain concentrations, respectively. The blue and red dashed lines represent the 90% prediction intervals in the plasma and brain, respectively. The green line represents the actual propofol dose rate (mg h^−1^).

The prediction-corrected visual predictive check (pcVPC) plot showed that the plasma and brain concentrations of propofol were in good agreement with the simulated concentrations in the models (Fig. 5). The median values of the bootstrap replicates and final parameter estimates were similar, indicating that the final parameters were appropriately estimated (Tables 1 and 2). Using a typical example of a propofol dosing schedule, the concentrations of propofol predicted using the Marsh, Schnider, and extended Marsh models are shown in Fig. 6. By adding the K_p_ value, the extended model provided a significantly different brain concentration than the Marsh and Schnider models. The model improvement due to the K_p_ was confirmed when analyzing the full dataset (57 patients) as well as the split datasets (Supplementary Table S3 and Fig. S2).

## Discussion

TCI administration of propofol using the Marsh or Schnider model is widely used for safe and stable anesthesia^1,2^. However, the accuracy of brain concentration has not been assessed in current PK models. Instead, the model has been verified by measuring plasma concentrations where plasma and effect-site concentrations have reached equilibrium^3–12^. This study found a significant positive correlation between the measured concentrations and the Marsh and Schnider model-predicted concentrations in the plasma, confirming the robustness of both models. The measured concentrations of propofol in the brain were higher than the effect site concentrations predicted using the Marsh and Schnider models or observed plasma concentrations.

A previous case study of five patients also reported higher brain concentrations of propofol than those predicted using the Marsh model^8^. Consistent with our findings, these results suggest that the concentrations at the site of action predicted using the Marsh and Schnider models did not correspond with brain concentrations. The brain distribution rate constant used in the Marsh and Schnider models is only k_1e_, which means that the predicted concentrations at the site of action cannot be higher than the plasma concentrations. Herein, we proposed extended models with a K_p_ value in addition to k_1e._ The K_p_ value enables an accurate description of brain concentrations higher than plasma concentrations. The K_p_ value functioned as a factor explaining retention in the brain. We used the same PK parameters as those in the Marsh model^3^ and found a good correlation between measured and predicted plasma concentrations. We considered that k_1e_ and K_p_ could be used to predict brain concentrations in models such as the Schnider^4,5^ and Eleveld^18,19^ models. In fact, the K_p_ values calculated using our new pharmacokinetic model based on plasma samples collected after the cessation of propofol administration were close. To establish extended models that predict brain concentrations, data on sequential brain concentrations, as well as those before the cessation of propofol administration, were used. Finally, the new dynamic models based on measured concentrations showed a more accurate prediction performance in the brain than the conventional models. This innovative study proposes new PK models with predicted brain concentrations.

In general, the tissue concentration of drugs can gradually increase, corresponding to an increase in the plasma concentration. Tissue concentrations slowly increase, especially in lipophilic drugs^20^. In previous reports, k_e0_ was reported^5,13^ as 0.26–0.456 min^−1^. In our new model, k_e0_ was 0.014 min^−1^, which was smaller than that previously reported. A small k_e0_ value indicates a slow drug transition between the plasma and tissues. The predicted concentrations of propofol using the Marsh, Schnider, and extended models are shown in Fig. 6. This shows how propofol is transferred from the blood to and retained in the brain. The previous models showed approximately the same values for plasma and brain concentrations at all times. Our new PK model showed that the process of transfer from the blood to the brain is sequential.

Regarding model validity, the plots of predicted and observed concentrations lay near the line of identity, and the plot of CWRES against population-predicted concentrations was evenly distributed. This indicated that the extended model and new model were unbiased and adequately described the variability. In addition, bootstrap statistics and pcVPC plots supported the acceptable performance of the final extended model and new model. Figure 4 shows that the regression line was close to the identity line. MdPE < 20% and MdAPE < 30% have been suggested to indicate clinically acceptable performance^21^. The MdPE and MdAPE values for the final extended model and new model were within an acceptable range. However, because a large performance error was observed in a few patients, further research is needed to assess error variability. In contrast, the median (range) MdPE values calculated using the Marsh model for the validating data were 8.6% (− 45.0 to 160%) for plasma and 206% (− 9.4 to 1170%) for brain concentration. Furthermore, the median (range) MdAPE values were 28.5% (0–160%) for plasma and 217% (9.4 to 1170%) for brain concentrations. Compared with the values calculated by the constructed model, the established model has better predictability than the previous model. These results indicate that the final extended model and new model were well established.

Our study has some limitations. First, lobectomy or uncapping margins around the brain tumor or epilepsy focus were used as normal brain samples. Although we collected data from non-pathological parts of the brain (lobectomy margin where signals on magnetic resonance imaging were normal), the possibility of the influence of the brain tumor or epilepsy focus in these areas remains. In addition, the tissue homogenate contains intra- and extra-cellular drugs and drugs in the blood vessels. Since propofol also significantly partitions into erythrocytes^22^, blood contamination is possible through both plasma and erythrocytes. However, even if a certain amount of blood is contaminated, it is unlikely to make a difference in the result that the concentration in brain parenchyma is mostly higher than the concentration in blood. Second, it has been reported that the clearance of propofol is accelerated in patients with frontal brain tumors^10^; however, whether this applies to patients with tumors in other areas is uncertain. Third, the extended model could not completely reduce the difference between the predicted and measured concentrations in the brain. There are still factors that cause inter-individual differences, including drug to drug interactions with antiepileptic drugs, expression levels, and genetic polymorphisms of pharmacokinetic genes. In this study, the predictability of brain concentrations was insufficient. To increase the estimates of inter-individual variability in brain concentrations, additional covariates should be identified in future studies. Fourth, we could not examine the relationship between the brain concentration of propofol and its efficacy, although adequate sedation targets were achieved. Future studies are required to develop a PK-PD model showing the relationship between brain concentration and clinical effects. Fifth, the plasma samples in our study were obtained from arteries similar to those in the Schnider model. However, the Marsh model was developed using venous samples. Differences in blood collection sites were not considered. Sixth, Figs. 1C and 5 showed that brain/plasma concentration ratio and brain propofol concentrations were increasing after 80 min. Because of the limited number of samples after 80 min, it is possible that a small number of high brain concentration samples affected the brain concentration curve. Finally, the measured concentrations were predicted using the Marsh and Schnider models. However, the predictive accuracy of the models was reported to be valid only after prolonged infusion and not at the initiation of infusion, where both models are fundamentally different^23^. Early blood samples should be collected to confirm the accuracy of the extended model and new model. Further studies are required to evaluate the clinical relevance of these models.

In conclusion, based on the Marsh model, this study established a new PK model for propofol using predicted brain concentrations from measured plasma and brain concentrations. The actual brain concentrations were higher than the current effect site concentrations predicted and plasma concentrations. The extended models can describe brain and plasma concentrations. This is the first step toward solving existing inaccuracies with current PK models and improving outcomes for patients underwent awake craniotomy. Future study is needed to show the applicability of the model results to other patient populations.

## Methods

### Approval for human experiments

This prospective study was approved by the Ethics Committee of the Kyoto University Graduate School of Medicine and Kyoto University Hospital (approval number: R1843, January 29, 2019) and was performed in accordance with the Declaration of Helsinki and its amendments. All eligible patients provided written informed consent before their registration or participation.

### Study design and participants

Ninety patients with brain tumors or brain lesions (epilepsy, cavernous hemangioma, or tuberculoma) underwent awake craniotomy at Kyoto University Hospital between May 2019 and June 2022. Eight patients did not consent to participate in the study. Initially, 82 patients were enrolled in this study. Twenty-five patients were excluded from the analysis because their plasma and brain sample quantities were too small for the analysis. Therefore, 57 patients were sequentially assigned to the model-building (29 patients, the first half) and validating group (28 patients, the second half) based on the order of enrollment. For each patient, most of plasma and brain samples (5–30 mg) were collected at five time-points: immediately before and at 5, 15, 25, and 75 min after the cessation of propofol administration to induce awakening. Depending on the surgical situation, some samples were collected at other times, and the time was recorded. Lobectomy or uncapping margins around the brain tumor or other sites were used as normal brain samples, which showed normal brain signals on preoperative magnetic resonance imaging. The following data were collected from electronic medical records: diagnosis, sex, age, propofol dosage, time of dosing and sampling, body weight, height, BMI, and clinical laboratory data, such as aspartate aminotransferase (AST), alanine aminotransferase (ALT), total bilirubin, albumin levels, and creatinine clearance (Ccr). Ccr was calculated using the Cockcroft–Gault equation with serum creatinine levels. Data on brain tumors and epileptic lesions were also collected.

### Sample preparation

The brain regions of the brain samples are as follows; 33 cases in the anterior cephalic lobe, 9 cases in the lateral cephalic lobe, 9 cases in the parietal lobe, 3 cases in the insular gyrus, 2 cases in the anterior lateral cephalic lobe, and 1 case in the hippocampus. The arterial sampling location is the radial artery. Samples were kept at − 30 °C in the operating room immediately after collection and then transferred to a − 80 °C freezer for storage. Plasma and brain samples were stored at − 80 °C until measurement. Brain samples were weighed, diluted 20 folds with saline, and homogenized. Plasma and homogenized liquids were used as samples. Calibration curves were prepared by spiking pool plasma (KAC Co., Ltd. Kyoto, Japan) with appropriate volumes of standard solutions to obtain calibration curve points equivalent to 0.5, 1, 3, 5, 7, 10, 15, 20, 25, and 30 mg mL^−1^ for propofol using Protein LoBind tubes (Eppendorf Co., Ltd., Tokyo, Japan). Subsequently, 50 µL of the sample and 150 µL of acetonitrile containing acetic acid (final acetic acid concentration: 0.1%) were vortexed and centrifuged (16,000×*g*, 10 min). The supernatant obtained after centrifugation was subjected to high-performance liquid chromatography. The mice brain samples of C57BL/6J mice (7-week, male) purchased from Japan SLC, Inc. (Shizuoka, Japan) were used as an alternative matrix to evaluate the matrix effect of the human brain samples, because blank human brain samples could not be obtained. The accuracy and precision of the matrix factor were ˂ 15% for all levels of quality control samples. All animal studies were conducted in accordance with the guidelines for animal experiments at Kyoto University. All protocols were approved by the Animal Research Committee of the Graduate School of Medicine, Kyoto University (Permission No. MedKyo21113).

Quantitative analysis was performed using a Prominence high-performance liquid chromatography system (Shimadzu Corp., Kyoto, Japan), according to a previous report^24^. Chromatographic separation was achieved using a Chemco Pak Liquid Chromatography Column (150 mm × 4.6 mm I.D.) (Chemco Plus Scientific Co., Ltd., Osaka, Japan) and maintained at 40 °C. The mobile phases comprised water containing acetic acid (35:0.1 v v^−1^) (mobile A) and acetonitrile (mobile B). The flow rate was maintained at 1.0 mL min^−1^. The detection wavelength was 276 nm for excitation and 310 nm for fluorescence, the analysis time was 15 min, and the injection volume was 2 μL. For quantitative analysis of all data, LC solution software (Shimadzu Corp.) was used.

### Simulation of plasma and brain concentrations using the Marsh and Schnider models

The plasma concentrations and effect compartments of propofol were calculated by simulation using the typical PK parameters of the Marsh^3^ or Schnider^4,5^ model. The parameters k_e0_ = 0.26 min^−1^ for the Marsh model^12^ and k_e0_ = 0.456 min^−1^ for the Schnider model^5^ were used to simulate the concentration in the effect compartment.

### PopPK modeling

All data on propofol administration rates (initiation to final points) and propofol concentrations (in the plasma and brain) at all points in the 29 patients in the model-building group were used to establish the PopPK model of propofol. PopPK analysis was conducted with the nonlinear mixed-effects modeling program NONMEM version 7.5.0 (ICON, Ellicott City, MD, USA) using the first-order conditional estimation (FOCE) method with interaction. Three-compartment with effect-compartment models and two-compartment with effect-compartment models were used for the structural PK model. Exponential error models were used for inter-individual variability. Intra-individual residual variability in serum and brain concentrations was compared between proportional, additive, and mixed (proportional and additive) error models.

The PK parameters of the brain compartments were also estimated. The differential equation describing the distribution of drugs in the brain compartment is as follows:

$$\frac{{dC_{brain} }}{dt} = k_{1e} *K_{p} *C_{central} - k_{e0}{\prime} *C_{brain}$$

where k_1e_ is the distribution rate constant from the central compartment to the brain compartment, K_p_ is the partition coefficient corresponding to the ratio of the brain to the plasma propofol concentration at steady state, C_central_ is the plasma concentration of propofol in the central compartment, k_e0_′ is the elimination rate constant from the brain compartment, and C_brain_ is the concentration of propofol in the brain. The k_1e_ value was calculated by dividing the intercompartmental clearance between the central and brain compartment (Q_4_) by V_1_. The k_e0_′ value was assumed to be the same as k_1e_, and the brain tissue’s density was considered^8^ 1 g mL^−1^. 

To assess the influence of continuous covariates, such as height, body weight, BMI, AST, and ALT, covariate analysis was performed using the following equation:

1 $${\text{Q}}_{4} = \, \uptheta_{1} *\left( {{\text{COV/COV}}_{{{\text{median}}}} } \right)^{\uptheta 2}$$

where θ_1_ is the mean parameter to be estimated, and θ_2_ is the factor contributed by the covariate. COV indicates the value for each patient, and COV_median_ is the median value of the clinical data. To assess the influence of sex, covariate analysis was performed using the following equation:

2 $${\text{Q}}_{4} = \, \uptheta_{1} *\left( {1 \, + \, \uptheta_{3} *{\text{sex}}} \right)$$

where sex is 1 for females and 0 for males, and θ_3_ is a factor contributing to sex.

The influence of each covariate on Q_4_ was evaluated according to the difference in the objective function value (OBJ) between the basic model and the model that included the covariate through forward stepwise inclusion. This result was verified using a backward stepwise elimination method. A p-value of < 0.05 (ΔOBJ > 3.84 with freedom of one assuming a chi-square distribution) in the forward inclusion and 0.005 (ΔOBJ > 7.88 with freedom of one assuming a chi-square distribution) in the backward elimination was considered statistically significant. The individually predicted PK parameters and concentrations were obtained by empirical Bayes estimation using the FOCE method. The raw data (all 57 patients) and the final NONMEM codes were included in the supplement files (Supplementary spreadsheet and codes).

### Model evaluation

The following diagnostic plots were used to evaluate model fitting: measured concentration versus PRED or IPRED, CWRES versus PRED, or time after cessation propofol to identify bias corresponding to model misspecification. To evaluate the central tendency predicted by the final model and the variability of the observed data, a pcVPC was performed using a simulation of 1000 datasets from the final model. The median and 90% prediction intervals of the simulated concentrations were plotted against the observed concentrations. The precision of the final model parameter estimates was assessed using asymptotic standard errors and nonparametric bootstrapping. Bootstrapping was conducted using 500 randomly sampled (replacement) replicates from 29 subjects in the original dataset. Model parameters were estimated for each bootstrap replicate using NONMEM.

Furthermore, the validity of the constructed model was evaluated using validating data that were not used for model construction. Briefly, the plasma and brain concentrations of propofol were estimated using a post hoc Bayesian method and compared with the observed concentrations for each patient in the validating cohort.

In addition, the predictive performance was evaluated by Varvel criteria^25^ using the following equations for MdPE and MdAPE:

$$PE_{ij} = \frac{{OBS_{ij} - PRED_{ij} }}{{PRED_{ij} }}*100\%$$

$$MdPE_{i} = median\left\{ {PE_{ij} , \;j = 1, \ldots ,N_{i} } \right\}$$

$$MdPAE_{i} = median\left\{ {\left| {PE_{ij} } \right|,\; j = 1, \ldots ,N_{i} } \right\}$$

where OBS_ij_ is the observed propofol concentration for the jth observation of the ith patient, PRED_ij_ is the PRED value corresponding to the observed data, and N is the number of patients. This quantifies the prediction bias and precision.

### Supplementary Information

Supplementary Information 1.

Supplementary Information 2.

## Data Availability

The datasets generated and/or analyzed during the current study are available from the corresponding author upon reasonable request.
