# Supplementary material for: Development of extended pharmacokinetic models for propofol based on measured blood and brain concentrations
Source: Sci Rep. 2024 Mar 15;14:6326. doi: 10.1038/s41598-024-56863-z (PMC10943190; doi:10.1038/s41598-024-56863-z)
Supplement: Supplementary file 1 — Supplementary Information 1. [file 41598_2024_56863_MOESM1_ESM.pdf]

Supplemental Information

Development of Extended Pharmacokinetic Models for Propofol Based on Measured Blood and Brain Concentrations

Masayoshi Kawata[1], Atsushi Yonezawa[1,2*], Yohei Mineharu[3,4], Kotaro Itohara[1], Toshiyuki Mizota[5], Yoshihiro Matsui[1,2], Takayuki Kikuchi[3], Yukihiro Yamao[3], Etsuko Yamamoto Hattori[3], Miho Hamada[5], Daiki Hira[1], Keiko Furukawa[6], Susumu Miyamoto[3], Tomohiro Terada[1], Kazuo Matsubara[1], Yoshiki Arakawa[3]

[1]Department of Clinical Pharmacology and Therapeutics, Kyoto University Hospital, 54 Shogoin Kawahara-cho, Sakyo-ku, Kyoto 606-8507, Japan

[2]Graduate School of Pharmaceutical Sciences, Kyoto University, 46-29 Yoshida Shimo-Adachi-cho, Sakyo-ku, Kyoto 606-8501, Japan

[3]Department of Neurosurgery, Kyoto University Graduate School of Medicine, 54 Shogoin Kawahara-cho, Sakyo-ku, Kyoto 606-8507, Japan

[4]Department of Artificial Intelligence in Healthcare and Medicine, Kyoto University Graduate School of Medicine, 54 Shogoin Kawahara-cho, Sakyo-ku, Kyoto 606-8507, Japan

[5]Department of Anesthesia, Kyoto University Hospital, 54 Shogoin Kawahara-cho, Sakyo-ku, Kyoto 606-8507, Japan

[6]Cancer Center, Kyoto University Hospital, 54 Shogoin Kawahara-cho, Sakyo-ku, Kyoto 606-8507, Japan

***Corresponding author:** Atsushi Yonezawa, Ph.D.

Department of Clinical Pharmacology and Therapeutics, Kyoto University

Hospital, 54 Shogoin Kawahara-cho, Sakyo-ku, Kyoto 606-8507, Japan

TEL number: +81-75-751-3581

FAX number: +81-75-751-4672

E-mail: ayone@kuhp.kyoto-u.ac.jp

Supplementary Table S1: Patient characteristics

	Model-building	Validation
Glioma/others	26/3	27/1
Male/female sex	16/13	19/9
Age, years	49 (13-76)	47 (17-77)
Height, m	1.66 (1.51-1.85)	1.66 (1.49-1.74)
Body weight, kg	62.5 (44.0-92.8)	64.3 (45.7-86.0)
BMI, kg m^{-2}	21.7 (16.9-33.1)	23.2 (17.9-32.4)
AST, U L^{-1}	20 (12-39)	21 (8-57)
ALT, U L^{-1}	22 (7-66)	38 (6-69)
ALB, g dL^{-1}	4.1 (2.8-4.7)	4.2 (2.9-4.9)
T-Bil, mg dL^{-1}	0.7 (0.3-1.3)	0.6 (0.3-1.1)
Ccr, mL min^{-1}	81.4 (49.9-150.5)	82.8 (45.5-127.2)

Each value represents a number or median (range). P-values were obtained using the Mann-Whitney and Fisher's exact tests for continuous and categorical values, respectively. Others included patients diagnosed with epilepsy, cavernous hemangioma, or tuberculoma.

Abbreviations: BMI, body mass index; AST, aspartate aminotransferase; ALT, alanine aminotransferase; ALB, albumin; T-Bil, total bilirubin; Ccr, creatinine clearance

Supplementary Table S2: Effect of tested covariates on the difference in OBJs

	Forward inclusion	Backward elimination
Tested covariates	ΔOBJ1	ΔOBJ2
Height	-0.40	
Body weight	-0.01	
BMI	-0.34	
SEX	0.00	
AST	-1.75	
ALT	-1.59	
ALB	-2.64	
T-Bil	-5.31	+5.31
Cre	-0.40	

Abbreviations: BMI, body mass index; AST, aspartate aminotransferase; ALT, alanine aminotransferase; ALB, albumin; T-Bil, total bilirubin; Cre, creatinine

Supplementary Table S3: Population pharmacokinetic parameters of propofol by full data (Extended Marsh model)

Parameter	Model-building		
	Estimates	%RSE	
k_{10} (min^{-1})	0.119		Fixed
k_{12} (min^{-1})	0.114		Fixed
k_{13} (min^{-1})	0.0419		Fixed
k_{21} (min^{-1})	0.055		Fixed
k_{31} (min^{-1})	0.0033		Fixed
V_1 (L kg^{-1})	0.228		Fixed
k_{1e} (min^{-1})	0.0123	24.1	
K_p	1.28	7.10	
Interindividual variability, CV%	Variance		
		%RSE	Shrinkage
IIV for k_{10}	19.3	10.0	12.8
IIV for k_{13}	45.7	30.4	45.4
IIV for K_p	37.1	22.2	30.4
Residual variability	Variance	%RSE	Shrinkage
Proportional error for plasma, CV%	18.1	14.0	19.5
Additive error for brain, µg/g	0.904	10.0	13.9

$k_{1e}=Q_4/V_1$

Abbreviations: 95% CI, 95% confidence interval; CV, coefficient of variation; IIV, interindividual variability; RSE, relative standard error; Q4, inter-compartmental clearance between the central and brain compartments

(A)

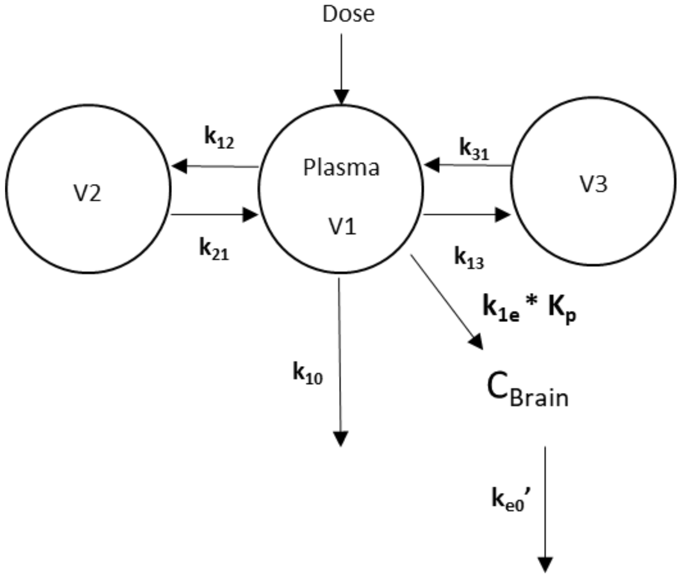

(B)

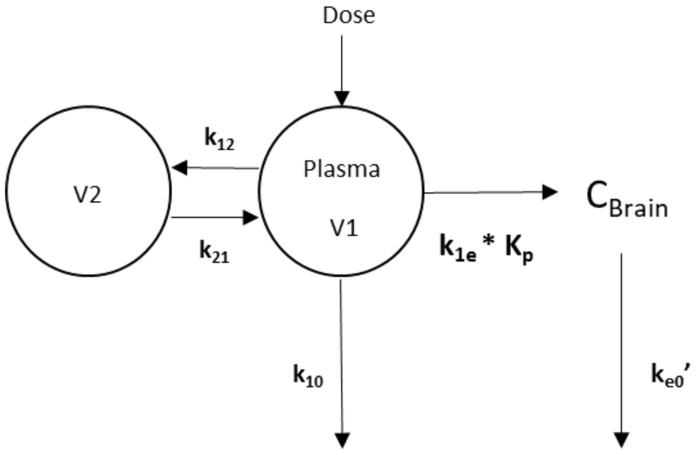

Supplementary Figure S1: Model structure.

A three-compartment and effect compartment model (A) is selected, corresponding to the Marsh model, where k_{1e} is the distribution rate constant from the central compartment to the brain compartment, K_p is the partition coefficient corresponding to the ratio of the brain to the plasma propofol concentration at steady state, k_{e0}’ is the elimination rate constant from the brain compartment, and C_{brain} is the concentration of propofol in the brain. A two-compartment and effect-compartment

model (B) is shown. The mean values of k_{10}, k_{12}, k_{13}, k_{21}, and k_{31} and the volume of the central compartment (V_1) are fixed with the mean parameter values used in the Marsh model. The parameters involved in the effect compartment and inter- and intra-variability are estimated. A proportional and additive error model for residual variability in serum and brain concentrations provides a better fit. For the predicted concentration of propofol in the brain, the inclusion of a K_p value for k_{1e} improves model fitting.

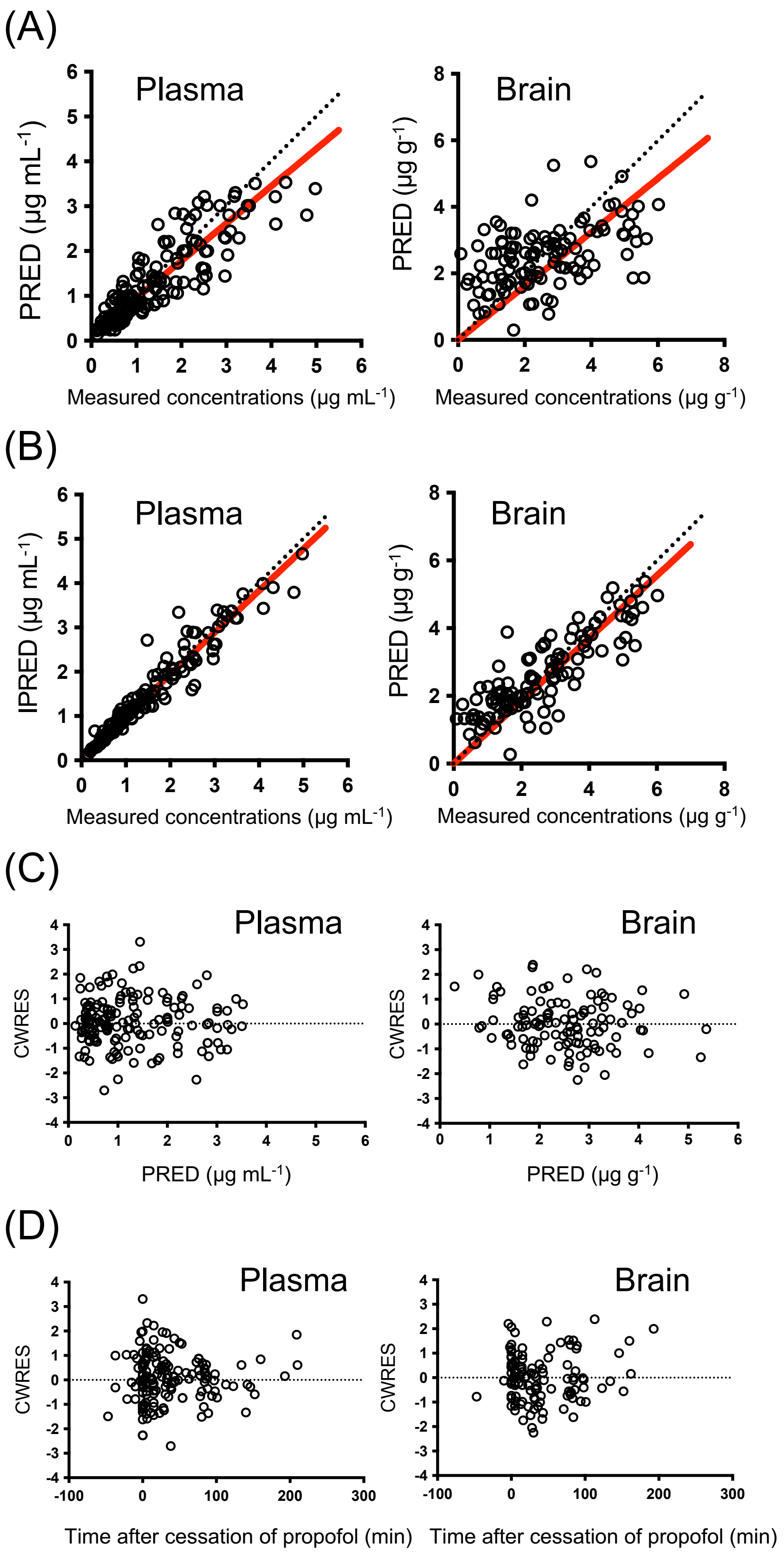

Supplementary Figure S2: Goodness-of-fit plots by full data for the extended propofol model

Measured concentrations versus population predictions (PRED) and individual predictions (IPRED) are shown in (A) and (B), respectively. Conditional weighted residuals (CWRES) versus PRED versus time after cessation of propofol are shown in (C) and (D). Empty circles represent patient data. There are 156 plasma concentrations and 112 brain concentrations in the 57 patients. The red line represents the regression line.

Supplementary code 1 Extended Marsh model

```
$PROB  Base model Propofol (3com:Marsh+Effect)
$INPUT ID TIME DOSE=AMT RATE CP=DV CMT MDV EVID
$INPUT HT BW BMI SEX AGE
$INPUT AST ALT ALB TBIL CRE
$DATA  220615_propofol4com.txt LRECL=800
$SUBROUTINES   ADVAN6 TOL=6
$MODEL COMP=(CENTRAL,DEFDOSE)
       COMP=(PERI1)
       COMP=(PERI2)
       COMP=(Effect)
$PK    k10=THETA(1)*EXP(ETA(1))
       k12=THETA(2)
       k13=THETA(3)*EXP(ETA(2))
       k21=THETA(4)
       k31=THETA(5)
       V1=THETA(6)*BW
       k14=THETA(7)
       Kp=THETA(8)*EXP(ETA(3))
       CL=V1*k10
       Q2=V1*k12
       Q3=V1*k13
       Q4=V1*k14
       V2=Q2/k21
       V3=Q2/k31
       S1=V1
       S2=V2
       S3=V3
$DES   DADT(1)=-k12*A(1)-k13*A(1)-k10*A(1)+k21*A(2)+k31*A(3)
       DADT(2)=k12*A(1)-k21*A(2)
       DADT(3)=k13*A(1)-k31*A(3)
       C=A(1)/V1
       DADT(4)=k14*Kp*C-k14*A(4)

$ERROR IF (CMT.EQ.1) THEN
       Y=F*(1+EPS(1))
       ELSE
       Y=F+EPS(2)
       ENDIF
       IPRED=F
$THETA 0.119 FIXED
$THETA 0.114 FIXED
$THETA 0.0419 FIXED
$THETA 0.055 FIXED
$THETA 0.0033 FIXED
$THETA 0.228 FIXED
$THETA (0,0.026)
$THETA (0,1)
$OMEGA 0.1 0.1 0.1
$SIGMA 0.1 0.1
$EST   METHOD=1 INTER  MAXEVAL=5000 PRINT=10
$TAB   ID CP TIME IPRED CWRES EVID CMT FILE=TABE006_2
$COV
```

Supplementary code 2 New model

```
$SIZES MAXFCN=10000000
$PROB  Base model Propofol (2com+Effect)
$INPUT ID TIME DOSE=AMT RATE CP=DV CMT MDV EVID
$INPUT HT BW BMI SEX AGE
$INPUT AST ALT ALB TBIL CRE
$DATA  230905_propofol3com.txt LRECL=800
$SUBROUTINES   ADVAN6 TOL=6
$MODEL COMP=(CENTRAL,DEFDOSE)
       COMP=(PERI)
       COMP=(Effect)
$PK    CL1=THETA(1)*EXP(ETA(1))
       V1=THETA(2)
       CL2=THETA(3)
       V2=THETA(4)
       k13=THETA(5)
       k10=CL1/V1
       k12=CL2/V1
       k21=CL2/V2
       Kp=THETA(6)*EXP(ETA(2))
       S1=V1
       S2=V2
$DES   DADT(1)=-k12*A(1)-k10*A(1)+k21*A(2)
       DADT(2)=k12*A(1)-k21*A(2)
       C=A(1)/V1
       DADT(3)=k13*Kp*C-k13*A(3)
$ERROR IF (CMT.EQ.1) THEN
       Y=F*(1+EPS(1))
       ELSE
       Y=F+EPS(2)
       ENDIF
       IPRED=F
$THETA 1.78
$THETA 41.1
$THETA 0.806
$THETA 123
$THETA 0.0149
$THETA 1.39
$OMEGA 0.0262 0.148
$SIGMA 0.045 0.772
$EST   METHOD=1 INTER  MAXEVAL=5000 PRINT=10
$TAB   ID CP TIME IPRED CL1 V1 EVID CMT FILE=TABB004_2.txt
$COV
```
